# A Statistical Study of Local Anesthesia in Dentistry

**Published:** 1902-03-15

**Authors:** Morris I. Schamberg


### A STATISTICAL STUDY OF LOCAL ANESTHESIA IN DENTISTRY.

BY MORRIS I. SCHAMBERG, D.D.S., M.D.

Read before the Pennsylvania State Dental Society, July 9, 1901.

Every innovation in medicine is met by more or less conservative opposition before it gains acceptance, and this attitude of conservatism is by no means to be deplored. There is, however, at times an excessive tendency to hastily decry or to pass unfavorable judgment upon measures which may ultimately be potent for good. In view of the fact that local anesthesia in dentistry has been adversely criticised by many men of prominence as teachers in the profession, and in view of its use in spite of this fact by many competent practitioners, I have made the effort to obtain an expression of opinion which would represent the prevailing sentiment of the profession on the question. I therefore addressed a circular letter, a little over a year ago, to about three hundred practicing dentists in different sections of the United States, requesting replies to the following questions:

1. Do you employ local anesthetics in your practice?

2. Kindly state what drugs or combination of drugs you use for this purpose, and also your method of employing them.

3. Have you observed any untoward effects, either constitutional or local, from their use?

4. What means, if any, do you find necessary to prevent post-operative swelling and sloughing?

5. Remarks or reports of interesting cases.

Copies of this letter and these questions were published in a few of the dental journals, so that any one desiring to record his experience and opinion might respond. Unfortunately there were but few replies through that source, and most of these, to the number of twenty-six, were laudatory of certain advertised anesthetic mixtures. Inasmuch as these replies were forwarded at the instance of commercial houses and with an evident intent to advertise their products, I have refrained from the publication of them in the report which I herewith present.

Only 132 legitimate replies were received. One hundred and two members of the profession stated that they used local anesthetics in their practice. Of the remaining thirty replies a few condemned the use of local anesthetics, while the rest merely stated that they did not use local anesthesia, inasmuch as they referred their dento-surgical cases to the specialist.

The replies show that cocain and eucain constitute the main ingredients of the injections used to benumb sensation in the mouth.

It may not be out of place to analyze the replies in reference to the preparations employed:

| | |
|---|---|
| Injections of aqueous solutions of cocaine | 23 |
| " " cocaine in combination with other drugs | 21 |
| " " aqueous solutions of eucaine | 20 |
| " " Dr. Hoff's formula (Parke, Davis & Co.'s tablets) | 12 |
| Ethyl chloride spray as refrigerant | 12 |
| Cocaine as a local application | 10 |
| Injections of chloretone | 4 |
| " " Wilson's anesthetic | 4 |
| " " Schleich's solution (infiltration) | 3 |
| " " nirvanin | 3 |
| " " alvatunder | 3 |
| " " Waite's anesthetic | 2 |

| | | |
|---|---|---|
| Injections of | Victor anesthetic | 1 |
| " | " Eureka anesthetic | 1 |
| " | " Antitortus anesthetic | 1 |
| " | " hydrogen dioxide | 1 |
| " | " alcohol, water, and salt | 1 |

In some instances untoward phenomena were reported, but not one case of a fatal termination has been recorded. Some of the replies in regard to methods employed for the purpose of obviating these conditions are decidedly interesting and instructive. While calculation shows that about 77 percent of those who responded use local anesthetics and counsel their employment, it must be remarked that this percentage is in all probability somewhat in excess of what it would be if an accurate census of the profession on this subject could be obtained. Those interested in the use of local anesthetics are naturally more prone to answer an inquiry in reference to this subject than those who do not employ them.

Again, it may be maintained that the aggregate number of letters sent out in this investigation was too small to warrant any definite conclusions.

Admitting the justice of these criticisms, the fact still remains as a result of this inquiry that a large number of practicing dentists of good repute are employing with satisfaction local anesthetics in the mouth. This is rather surprising in view of the fact in that many of the prominent dental colleges of the country the students receive little or no instruction concerning local anesthetics, and are frequently admonished against their use.

Let us now consider the chief objections which have been urged against the use of local anesthetics in dentistry. They may be stated as follows:

1. *Unsatisfactory anesthesia.* It has been claimed by some practitioners that local anesthesia is unsatisfactory because of the incomplete loss of sensation produced. This result may be attributed to imperfect technique. It should be remembered that the administration of chloroform, ether, or nitrous oxide is as unsatisfactory as the use of cocaine, eucaine, or the ethyl chloride spray in the hands of an inexperienced operator. Too small a quantity of the anesthetic will render the operation painful, whereas too much will endanger the patient's condition. How often, with as harmless an anesthetic as nitrous oxide, will the timid administrator proceed with the operation in hand before the patient is fully anesthetized. It may be justly claimed, then, that the poor results achieved by dentists with anesthetics, both general and local, are largely due to the fact that many of the dental schools do not give this subject the consideration it requires. With these facts in mind it must be evident that it matters little whether we use general or local anesthetics, our results will not be perfect unless we are skilled in their manipulation.

2. *Post-operative swelling, sloughing, and necrosis.* Post-operative swelling, sloughing, and necrosis are said to frequently follow the use of local anesthetics. Too often sequence is confounded with consequence. Many of those present have doubtless seen cases of swelling, sloughing and necrosis occur after extraction when no local anesthetic had been employed. These conditions are particularly prone to develop in individuals debilitated by acute disease or suffering from some constitutional effection such as anemia, tuberculosis or syphilis. Local causes, such as traumatism incident to difficult extraction, infection from pre-existing abscesses and the accidental presence of virulent germs in the mouth, often play an important *role.*

It must be admitted that the improper use of local anesthetics may determine one of the accidents above referred to. It is quite conceivable that fluid injected beneath the periosteum may interfere with the nutrition of the bone sufficiently to give rise to necrosis. Again, improper aseptic technique as regards instruments, solution, or the mouth, may give rise to infection of the tissues with destructive micro-organisms.

Admitting that there may be some influence following the use of local anesthetics tending to temporarily lower the resisting power of the tissues to which they have been applied, yet danger can be forestalled by the vigorous application of antiseptics before, during and after the operative procedure.

But it is a question whether these dangers have not been overestimated. Solutions of cocaine and eucaine are used beneath the skin, upon the mucous membranes of the nose and eyes, and even injected directly into the spinal canal, without danger to the patient so long as proper precautions are taken. Experience with the use of cocaine upon such a delicate structure as the spinal cord is sufficient proof of the fact that the drug *per se* exerts no injurious influence upon nerve tissue. Therefore necrosis and swelling must be explained upon some other grounds.

In the production of analgesia by refrigeration of the part, destruction of the tissue might result as does chilblain from an excessive exposure to cold. With due caution, however, this can be prevented.

3. *The danger of systemic poisoning.* It can not be denied that the use of cocaine is attended with some risk owing to the varying degree of susceptibility of different individuals to this drug. Ordinarily, however, the amount necessary to produce local anesthesia is not sufficient to produce untoward symptoms. In cases of poisoning from small doses, while alarming symptoms may be exhibited, these usually subside without injury to the patient. Fatal cases of cocaine poisoning are rare. If cocaine can be injected beneath the skin and beneath the dura mater of the spinal cord without harm to the patient, there is no reason why it can not be used in the mouth. Too frequently the operator becomes alarmed by manifestations which are referable to psychic disturbances rather than to the influence of the drug upon the vital centers. The dose of the drug used is, of course, a matter of great importance. Ordinarily a one-eighth grain of cocaine hydrochlorate may be injected without much liability to toxic symptoms. If one desires, however, to obviate the danger of systemic poisoning, he may substitute for cocaine the less toxic drug, beta-eucaine. The employment of anesthetic mixtures the active strength of which is unknown to the dentist is to be strongly deprecated.

I trust that the presentation of this paper will evoke a free expression of opinion. We are all in search of the truth, and any information which will shed light on the advantages or disadvantages of local anesthesia should not be withheld. Dentists should be encouraged to publish unfavorable results and complications as well as favorable reports, and these should be dispassionately adjudged by the profession.

Personally I only employ local anesthetics when for some reason nitrous oxide will not answer my purpose. I believe local anesthetics should have a distinct place in dental surgery. Properly employed in judiciously selected cases, they give results satisfactory alike to the dentist and the patient. They do not supplant the use of nitrous oxide and other general anesthetics, but merely supplement them.—*Dental Cosmos.*

